# Research on the Method of Imperfect Wheat Grain Recognition Utilizing Hyperspectral Imaging Technology

**DOI:** 10.3390/s24196474

**Published:** 2024-10-08

**Authors:** Hongtao Zhang, Li Zheng, Lian Tan, Jiapeng Yang, Jiahui Gao

**Affiliations:** College of Electrical Engineering, North China University of Water Resources and Electric Power, Zhengzhou 450046, China; zl2711673806@163.com (L.Z.); tanlianedu@126.com (L.T.); 17513364216@163.com (J.Y.); gjh13283826970@126.com (J.G.)

**Keywords:** imperfect grains of wheat, hyperspectral imagery, support vector machines, convolutional neural networks, MobileNet V2

## Abstract

As the primary grain crop in China, wheat holds a significant position in the country’s agricultural production, circulation, consumption, and various other aspects. However, the presence of imperfect grains has greatly impacted wheat quality and, subsequently, food security. In order to detect perfect wheat grains and six types of imperfect grains, a method for the fast and non-destructive identification of imperfect wheat grains using hyperspectral images was proposed. The main contents and results are as follows: (1) We collected wheat grain hyperspectral data. Seven types of wheat grain samples, each containing 300 grains, were prepared to construct a hyperspectral imaging system for imperfect wheat grains, and visible near-infrared hyperspectral data from 2100 wheat grains were collected. The Savitzky–Golay algorithm was used to analyze the hyperspectral images of wheat grains, selecting 261 dimensional effective hyperspectral datapoints within the range of 420.61–980.43 nm. (2) The Successive Projections Algorithm was used to reduce the dimensions of the 261 dimensional hyperspectral datapoints, selecting 33 dimensional hyperspectral datapoints. Principal Component Analysis was used to extract the optimal spectral wavelengths, specifically selecting hyperspectral images at 647.57 nm, 591.78 nm, and 568.36 nm to establish the dataset. (3) Particle Swarm Optimization was used to optimize the Support Vector Machines model, Convolutional Neural Network model, and MobileNet V2 model, which were established to recognize seven types of wheat grains. The comprehensive recognition rates were 93.71%, 95.14%, and 97.71%, respectively. The results indicate that a larger model with more parameters may not necessarily yield better performance. The research shows that the MobileNet V2 network model exhibits superior recognition efficiency, and the integration of hyperspectral image technology with the classification model can accurately identify imperfect wheat grains.

## 1. Introduction

Wheat, as the primary food crop in many people’s lives, holds a pivotal position in China’s agricultural production, circulation, consumption, and other related aspects [1]. As one of the most important criteria for evaluating wheat quality, the content of imperfect grains in wheat has research value that cannot be ignored. Finding the most effective detection method has been a key concern for food scientists globally. Thanks to advancements in computer image technology, pattern recognition, machine vision, near-infrared spectral analysis, and hyperspectral imaging, featuring rapid non-destructive identification, these have been widely adopted for seed classification and recognition. Hyperspectral imaging integrates near-infrared spectroscopy with image technology, and it boasts a unique advantage: it can capture both the image and spectral data of the subject. The acquisition of this dual information provides more comprehensive and in-depth data support than a single information source. Traditional detection methods often require image acquisition and spectral analysis to be performed separately, whereas hyperspectral imaging can accomplish these two tasks simultaneously during a single detection process, greatly enhancing both detection efficiency and accuracy [2,3]. Hyperspectral imaging technology has been widely used in the field of agriculture due to its advantages of fast detection speed, low cost, no damage to samples, simple operation, and so on.

In 2009, Singh et al. identified insect-infested wheat grains using hyperspectral images in the range of 1101.69–1305.05 nm, achieving a recognition rate of 85%. Employing a quadratic discriminant analysis classifier, they achieved a recognition rate of 96.4% for insect-infested grains, while using linear discriminant analysis, they achieved 97.3% for moldy grains [4,5,6]. In 2016, Liang Kun et al. established three recognition models for Fusarium head blight grains in wheat, all with recognition rates exceeding 90% [7]. In 2017, Yu Chongchong et al. collected hyperspectral images of four types of imperfect grains and established a Convolutional Neural Network (CNN) to recognize images under 30 wavebands, achieving a recognition rate of 99.98% on the test set [8]. Dong Jingjing et al. collected hyperspectral images of four types of imperfect wheat grains and established Support Vector Machine (SVM) classification models using spectral and image features separately [9]. Yu Le et al. collected hyperspectral data for four types of imperfect wheat grains in the range of 493–1106 nm, selected 24 wavebands from 116, and established a CNN model that achieved recognition rates exceeding 92% for three types of imperfect grains [10]. In 2019, Liu Huan et al. collected hyperspectral data for four types of imperfect wheat grains in the range of 862.9–1704.2 nm, used DPLS and OLDA methods to reduce the dimensionality of spectral features, and achieved a recognition rate of 97.8% [11]. In 2018, Hao Chuanming et al. fused hyperspectral images with high-resolution images of imperfect wheat grains and improved recognition accuracy by using a VGG network to recognize the fused images [12]. Liu Shuang et al. collected hyperspectral images of wheat grains in the range of 469–1082 nm and established an SGSPA-SVM classification algorithm to recognize the images, achieving a recognition accuracy of 98% on the test set [13].

In the present study, when using hyperspectral analysis to detect imperfect particles, the selected experimental objects are mostly limited to several types of imperfect particles, and do not cover all kinds of imperfect particles. In addition, regarding the germinating wheat grain, when it has just germinated, the embryo will bulge, but no obvious bud will have been formed. However, most studies tend to use wheat grains in the late germination stage when selecting germinated grains as samples. If these germinated grains can be successfully detected at the early stage of germination, it will help to reduce the loss caused by germinated grains.

This paper mainly studies the classification of imperfect wheat grains based on hyperspectral images. There are six kinds of imperfect grains, including insect-eaten wheat grains, gibberella grains, black embryo grains, broken grains, germinated grains, and moldy grains, in addition to perfect grains, making a total of seven wheat grain categories. The rapid and non-destructive identification of imperfect wheat grains can be realized through the combination of near-infrared hyperspectral technology and convolutional neural network technology.

In order to study the recognition of imperfect wheat grains from hyperspectral images, this study takes the wheat grain variety Bainong 207 as the research object and establishes Particle Swarm Optimization for optimizing Support Vector Machines (PSO-SVM), CNN, and the MobileNet V2 recognition model. Additionally, this study uses the Successive Projections Algorithm (SPA) to extract characteristic wavelengths.

## 2. Materials and Methods

### 2.1. Materials

This article defines wheat as one of two categories, categorized into perfect grains (normal, undamaged wheat) and imperfect grains, which refer to six kinds of damaged wheat. These six categories are broken wheat, germinated wheat, moldy wheat, insect-eaten wheat, black embryo wheat, and gibberella wheat. To accurately identify perfect grains and imperfect grains, we collected perfect grains and the six kinds of imperfect grains, making a total of seven kinds of wheat grains.

The damaged grains were obtained by artificial destruction, while the insect-eaten grains, germinated grains, and moldy grains were obtained through specific treatments. The wheat variety selected for the experiment/study was Bainong 207. Firstly, impurities and unsatisfactory grains were filtered out using a sieve, and perfect wheat was selected as the test sample. The diameter of the grain of Bainong 207 wheat is usually between 6 and 8 mm. The size of the unfilled particles is usually smaller than that of the perfect particles. Therefore, in this study, a sieve with a pore size of 6 mm was selected to eliminate larger impurities and some unsuitable particles. Following this initial screening, professionals conducted further assessments. Using this approach, we could efficiently identify high-quality wheat for use as test samples.

This screened wheat was then divided into three groups. One portion was combined with insect-eaten grains and placed in Culture Bottle No. 1, another was mixed with moldy grains and put into Culture Bottle No. 2, and the final portion remained unchanged in Culture Bottle No. 3.

Culture Bottle No. 1 was incubated at room temperature (25 °C) without any additional treatment. After 7 days, the wheat was taken out and the insect-eaten wheat was selected. Culture Bottle No. 2 was placed at a constant temperature and a humidity incubator was set at 30 °C and 80% humidity. After 7 days, the wheat was taken out and moldy wheat was chosen from the mixture.

The wheat from Culture Bottle No. 3 was soaked in water at 30 °C for 12 h. After soaking, it was returned to the culture bottle, left at room temperature (25 °C), and the wheat was sprayed with water every other hour. Two days later, germinated wheat was selected from the bottle. At this stage, the wheat embryo was slightly raised, and sprouts/germ were just beginning to appear, indicating the early phase of wheat germination. This germinating wheat was taken as the experimental sample.

**Figure 1:**
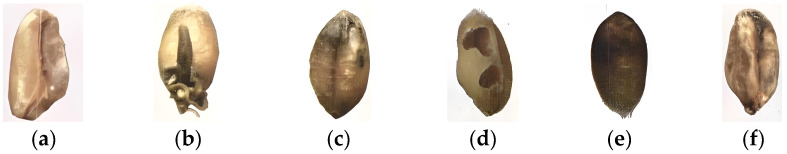
Hyperspectral images of seven types of wheat grains. (**a**) Broken wheat; (**b**) germinated wheat; (**c**) moldy wheat; (**d**) insect-eaten wheat; (**e**) black embryo wheat; (**f**) gibberella wheat.

Two types of diseased grains, black embryo grain and gibberella grain, are characterized by fungal infections during wheat growth, and they naturally arise from these infections. These two types of imperfect grains were obtained from the Wheat Research Institute of Henan Academy of Agricultural Sciences. Finally, 300 perfect grains and 300 imperfect grains of each type (broken wheat, germinated wheat, moldy wheat, insect-eaten wheat, black embryo wheat, and gibberella wheat) were used as test samples. The typical state of the imperfect grains is shown in Figure 1.

### 2.2. Instruments

**Figure 2:**
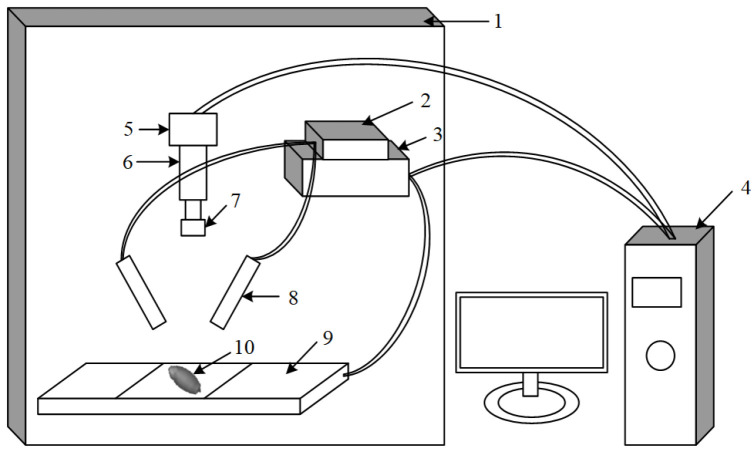
Visible hyperspectral imaging system. (1) Closed box; (2) light source; (3) displacement stage controller; (4) computer; (5) visible light camera; (6) imaging spectrometer; (7) lens; (8) glass fiber linear lamp; (9) displacement stage; (10) wheat grain.

A visible light hyperspectral image acquisition system has been constructed, consisting primarily of a spectral imaging module, a lighting module, a displacement module, a computer, and an enclosed box. The overall system structure is depicted in Figure 2.

The Spectral Imaging module includes a CCD camera (DL-604M, Andor, Ireland), a spectrometer (V10E, Specim, Oulu, Finland, Spectral Imaging Ltd.), and a lens (OLE23, Schneider, Rueil-Malmaison, France). When collecting images, we turned on the light source and placed the wheat grains under the light. The spectrometer slit captured the image of the instantaneous field of view; then, the grating and prism dispersed the instantaneous image perpendicular to the direction of the sample strip, finally forming the image on the image plane of the CCD camera.

The lighting module consists of two parts: a 150 W DC adjustable tungsten halogen lamp light source (IT 3900e, Illumination Technology Inc., New York, NY, USA) and a glass fiber linear lamp (Illumination Technology Inc., East Syracuse, New York, NY, USA).

The shift module includes two parts: a displacement table (IRCP0076-1COMB from Beijing Optical Instrument Factory, Beijing, China) and a controller (SC100 from Beijing Optical Instrument Factory).

The computer module is configured with an Intel Core i7-4790 CPU, 32 GB RAM, 500 GB hard disk, and an independent 256 MB graphics card. The inner wall of the enclosed box is sprayed black in a high-temperature electrostatic environment, eliminating the influence of static electricity. Several other modules are placed inside the enclosed box, where the spectral camera, light source, and other components can be freely adjusted. The two doors of the enclosed box can be easily opened and closed.

### 2.3. Acquisition of the Hyperspectral Images

Prior to gathering spectral data on wheat grains, multiple tests were conducted. It was determined that the optimal image quality was achieved when the exposure time was set to 3 ms and the displacement table moved at a speed of 4.012 mm/s [14].

When the displacement table shifts, the wheat’s position on the stage alters due to inertia. Therefore, to prevent movement, the wheat grains are secured onto black electrical tape before imaging. The reflectivity of the black electrical tape is low.

Furthermore, the hyperspectral image underwent calibration to negate the effects of current noise, with the image transformation outlined in Formula (1).

(1) $$R=\frac{I-B}{W-B}$$

where I represents the imperfect particle image collected when the light source is turned on, *B* is the calibration image in the full black environment, *W* is the full white calibration image obtained by scanning the white correction plate, and *R* is the converted image.

**Figure 3:**
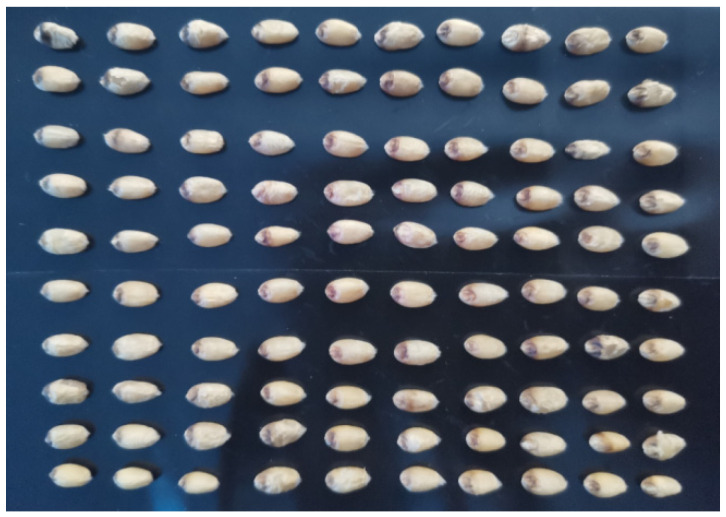
Arrangement of wheat grains.

To ensure data validity, this study captured 300 hyperspectral images, including those of pristine particles and six types of flawed particles. Given the vast amount of wheat grain data needed, we grouped 100 grains together in a way that did not compromise subsequent image analysis, streamlining the image capture process. These 100 grains are arranged in a 10 × 10 grid, ensuring no overlap and minimal spacing between grains, as illustrated in Figure 3. The displacement table is then moved to the hyperspectral camera’s imaging area. To maintain a constant ambient temperature and prevent moisture changes within the wheat grains, the room air conditioner is adjusted to a constant room temperature. The visible hyperspectral camera captures a spectrum ranging from 374.28 nm to 1019.91 nm, across 302 bands. When collecting images, the displacement table moves from left to right, scanning one straight line at a time. After scanning n straight lines, a hyperspectral data cube of 1600 × n × 302 is obtained. Here, 1600 denotes the pixel resolution of the image width that the camera is capable of capturing in a single scan. This specific value is determined by the camera’s hardware design and its configuration during the imaging process.

### 2.4. Spectral Processing and Sample Division

**Figure 4:**
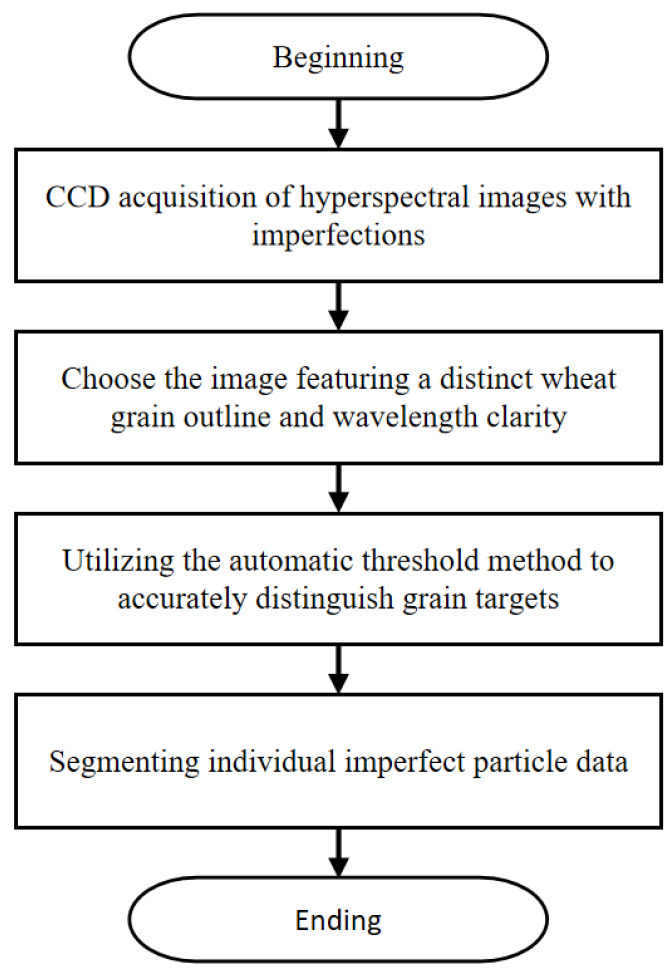
Flow chart of hyperspectral data processing.

The raw data obtained from the hyperspectral image acquisition system are raw files, which cannot be directly processed by MATLAB. The original file format is converted into a MAT file that can be read by MATLAB using ENVI. Due to defects in the manufacturing process, there are some bad points on the visible CCD during shooting, resulting in noise in the obtained hyperspectral data. In addition, 100 grains are taken as a group during shooting. To facilitate the subsequent identification of imperfect grains, the collected hyperspectral data need to be segmented into individual grain data. Therefore, the collected hyperspectral data require preprocessing. The specific workflow is shown in Figure 4.

**Figure 5:**
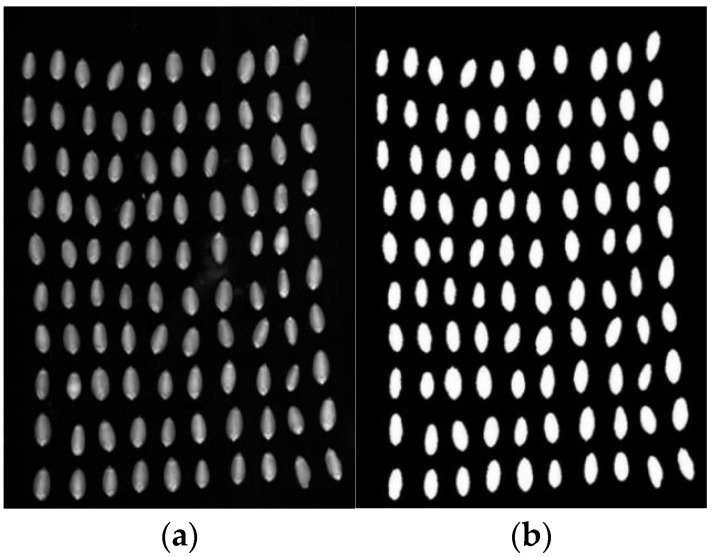
Hyperspectral image at 736.59 nm. (**a**) Hyperspectral image; (**b**) binary image.

**Figure 6:**
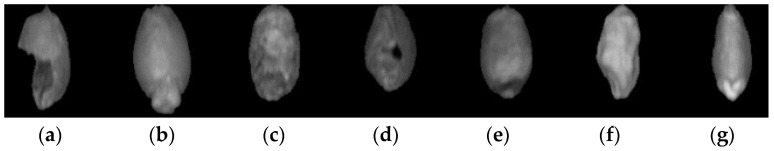
Hyperspectral images of seven types of wheat grains. (**a**) Broken wheat; (**b**) germinated wheat; (**c**) moldy wheat; (**d**) insect-eaten wheat; (**e**) black embryo wheat; (**f**) gibberella wheat; (**g**) normal wheat.

(1) After acquiring hyperspectral data using the hyperspectral camera, the raw data are segmented by ENVI into a hyperspectral data cube with dimensions of M1 × M2 × 302, where M1 represents the width of the segmented data, M2 signifies the height of the segmented image, and 302 denotes the total number of wave bands.

(2) Following segmentation and transformation, wheat grain hyperspectral images undergo thresholding segmentation using the maximum between-class variance method, commonly known as Ostu’s method. In this approach, the segmented target is considered the foreground, while the remaining areas are treated as the background. The segmentation of the target is achieved by maximizing the variance between the foreground and background. The hyperspectral data cube was opened using MATLAB, and a detailed analysis was conducted on the images across 302 bands. Among these, the images captured at a wavelength of 736.59 nm (corresponding to band 173) exhibited relatively clear visuals, effectively distinguishing imperfect grains from the background. Consequently, this specific wavelength was chosen for image segmentation. The regions of interest (ROI), specifically the areas containing wheat, were extracted from the broader hyperspectral dataset through segmentation. This process ensured the retention of all pertinent wheat data and filtered out impurities and other undesirable elements. Subsequently, an optimal threshold was selected to segment the entire hyperspectral dataset using the Otsu method, generating a hyperspectral binary image. In this binary representation, the wheat grain regions appear white, standing out distinctly from the black background. Figure 5 displays both the hyperspectral image of a set of wheat grain samples captured at 736.59 nm and the corresponding segmented binary image.

(3) After the segmentation and transformation of the hyperspectral data, the wheat grains are organized in ascending order from bottom to top and from right to left. The center coordinate of each wheat grain is determined based on its serial number. By expanding from this center coordinate, the edge of each wheat grain is identified. Subsequently, the data pertaining to each wheat grain are preserved, resulting in single-grain data. Refer to Figure 6 for hyperspectral images of seven wheat grains.

### 2.5. Modeling Method

POS-SVM: This exhibits high classification accuracy for small sample sizes and high-dimensional data. With strong generalization capabilities, it can enhance the performance of models, as evidenced by studies [15,16,17]. However, it is sensitive to noise, which may affect classification performance. Given the focus of this study on scenarios with limited data samples or complex feature spaces, POS-SVM is a favorable choice due to its ability to efficiently process high-dimensional data while maintaining high accuracy.

CNN: This boasts automatic feature extraction capabilities, making it particularly suitable for image processing and computer vision tasks. Leveraging GPU acceleration for efficient parallel computing, CNN significantly accelerates the training process. However, it requires substantial training data to avoid overfitting, and deep networks often take a long time to train with poor interpretability. Since this study involves complex image tasks requiring automatic feature extraction, CNN excels in this area.

MobileNet V2: When performing image classification and object detection on mobile devices [18], this demonstrates high accuracy, rapid inference speed, and robust real-time capabilities [19,20,21]. Nevertheless, for complex problems, its accuracy is limited and its deep feature extraction capabilities are weaker. Further adjustments may be necessary for specific scenarios. Since this study involves image classification, MobileNet V2 is a suitable choice.

## 3. Results and Discussion

### 3.1. Spectral Preprocessing and Sample Division

**Figure 7:**
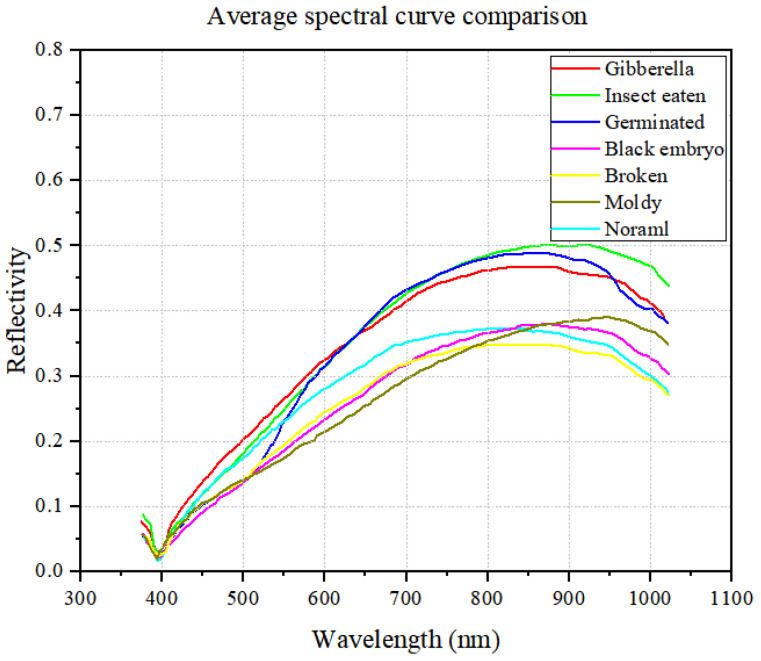
Average spectral reflectance curves of seven types of wheat grains.

By averaging the spectral data of various wheat types, based on the spectral reflectance of each wheat type across 302 bands at wavelengths ranging from 374.28 nm to 1019.91 nm, the average spectral reflectance curve for wheat grain is derived, as illustrated in Figure 7.

Based on Figure 7, it is evident that the average spectral reflectance curves for seven types of wheat grains exhibit similar patterns across the range of 374.28–1019.91 nm, initially increasing and then decreasing. However, subtle variations exist among them. Notably, the spectral curves for insect-eaten grains, germinated grains, and gibberella grains stand out compared to those of the other four wheat types. The presence of wormholes in wheat grains, often caused by pests like bark beetles and rice weevils, creates significant disparities in spectral characteristics compared to flawless grains. Gibberella grains, due to their lack of moisture, appear white and shriveled, resulting in a smaller overall volume than perfect grains. Germinated grains, which sprout in the embryo, consume starch and protein, leading to distinct spectral properties. Moldy grains undergo surface changes due to fungal erosion, while black embryo grains turn dark brown or black because of fungal infection. Broken grains, as imperfect wheat grains, also exhibit spectral differences compared to flawless grains; however, the spectrum of these wheat grains is less different from that of the perfect grains.

**Figure 8:**
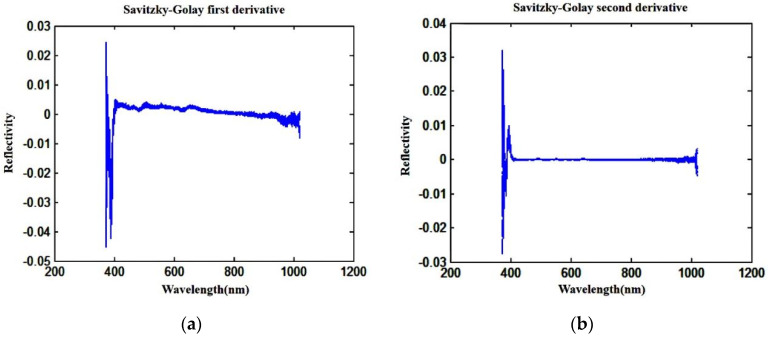
Derivation results of hyperspectral data. (**a**) Savitzky–Golay first derivative; (**b**) Savitzky–Golay second derivative.

Spectral data can reflect the chemical composition and component concentration in wheat grains, and are also affected by physical information such as grain surface texture and density. By processing the spectrum, the problems caused by the response of the visible light spectrum to the instrument and the change in the test environment can be eliminated, thereby improving the stability of the model. The first-order and second-order derivatives calculated using the Savitzky–Golay (S-G) algorithm were used to process the original data [22], and the derivation results are shown in Figure 8.

During the collection of wheat grain spectral data, factors such as scattering information, noise, and baseline drift cause fluctuations in the spectral curve, particularly in the 374.28–420.61 nm and 980.43–1019.91 nm bands. To guarantee the precision of the subsequently established model, these two bands are excluded, and only the spectral data from 420.61 to 980.43 nm are utilized for creating the imperfect wheat grain recognition model.

### 3.2. Spectral Data Dimensionality Reduction

The hyperspectral data collected for wheat grains, spanning the wavelength range of 420.61–980.43 nm, consist of a total of 261 dimensions. However, there is noticeable information redundancy among adjacent bands, which can potentially hinder the training efficiency and recognition accuracy of the recognition model, ultimately failing to meet the practical detection needs for imperfect grains. Consequently, it becomes imperative to reduce the dimensionality of the 261-dimensional spectral data. To achieve this, this study used the SPA to effectively reduce the dimensionality of the spectral data for wheat grains across the 261 bands within the 420.61–980.43 nm range.

**Figure 9:**
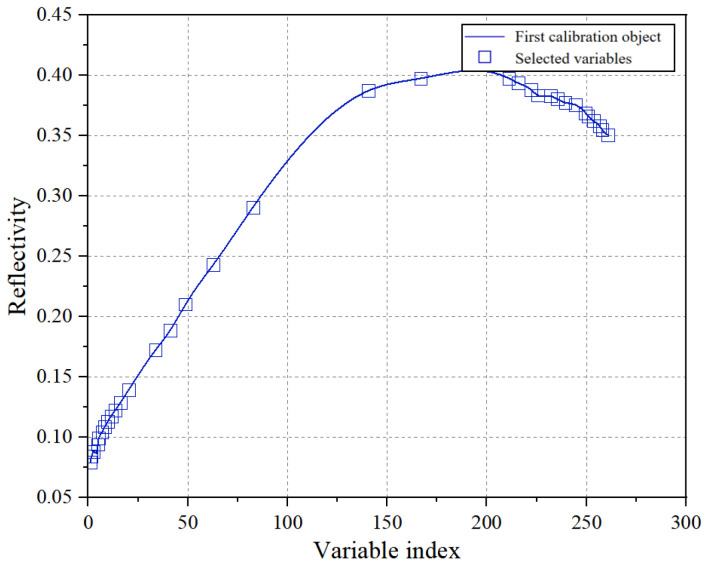
Feature selection results of SPA.

SPA initially selects a wavelength at random from the entire spectrum. It then computes the projection between this chosen wavelength and all others. This process is repeated cyclically for all wavelengths, comparing the results of each projection. The wavelength combination yielding the highest projection value is deemed the optimal one [23]. The SPA feature selection outcomes are illustrated in Figure 9. Following a multivariate linear analysis, the best result is determined. From the original 261 dimensions, 33 were carefully selected, representing characteristic bands at 420.61, 422.64, 428.75, and 432.83 nm. The feature space is subsequently constructed using these refined 33-dimensional spectral data.

### 3.3. Modeling Results

#### 3.3.1. PSO-SVM Model

**Figure 10:**
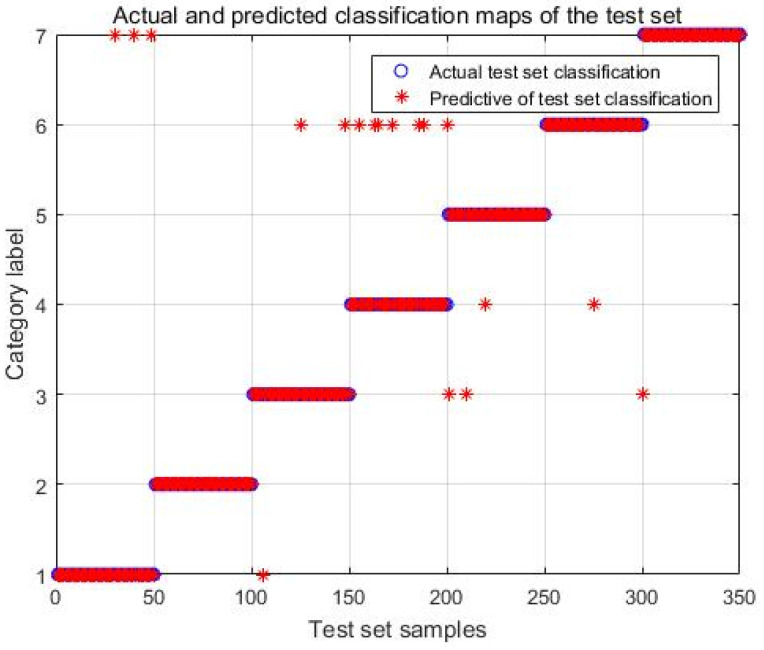
Classification results of spectral features optimized by SPA.

During the training of the SVM model, the penalty factor C (ranging from 0 to +∞) and the RBF kernel function parameter g (variance) emerge as critical factors influencing the recognition rate. Exploring intelligent algorithms to fine-tune these two parameters can significantly enhance the model’s recognition capabilities. Notably, when c = 46.48 and g = 0.9775, the model attains optimal performance. Under these conditions, the overall recognition rate for seven types of wheat grains reached 93.71%, with 328 out of 350 grains thus correctly identified. However, Figure 10 reveals apparent recognition errors, particularly for broken and insect-eaten grains (the grains are ordered from left to right as follows: broken grain, germinated grain, moldy grain, insect-eaten grain, black embryo grain, gibberella grain, and perfect grain).

#### 3.3.2. CNN Model

**Figure 11:**
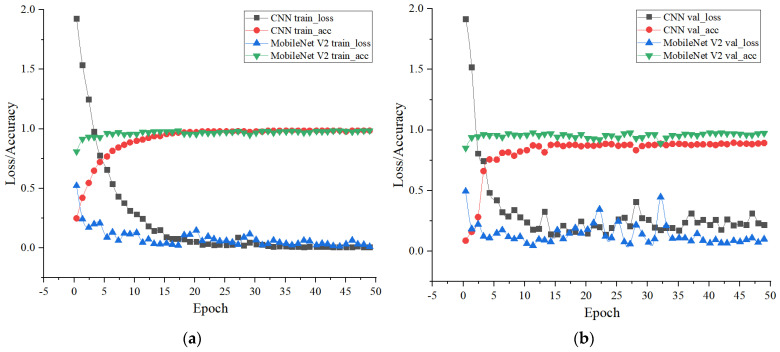
CNN and MobileNet V2 model recognition results. (**a**) Training accuracy and training loss; (**b**) Verification accuracy and verification loss.

Before establishing the model, the dataset is first set up. The optimal classification wavelength is 647.57 nm, which is the first factor of the first principal component. The images under this wavelength are saved, including 300 images of each type of wheat grain. In order to increase the amount of data, 568.36 nm images corresponding to the first factor of the second principal component and 591.78 nm images corresponding to the first factor of the third principal component are selected. As a result, there are 900 images of each type of wheat grain. Among these, 250 images of each type are randomly drawn and designated as the training set and 50 as the validation set, and an additional 50 images at 649.73 nm (corresponding to the second factor of the first principal component) are chosen as the test set. The final dataset consists of 5250 training images, 1050 validation images, and 350 test images. The convolutional neural network is trained in 50 steps, with the training outcomes illustrated in Figure 11.

During the model training process, it is observed that the initial learning rate significantly affects the model’s performance. By setting an appropriate learning rate, the model can converge towards the optimal solution more efficiently. In this instance, the initial learning rate was set to 0.0005. The training results indicate that the model stabilizes within 10 training steps. Remarkably, after just 50 steps of training, the model achieved a training accuracy of 98.87%, with a training loss of less than 0.1, nearing zero. However, while the recognition rate of the validation set reached 95.22%, the validation loss hovered around 0.3, and did not continue to decrease.

#### 3.3.3. MobileNet V2 Model

The MobileNet V2 model has been established, utilizing the same training and validation sets as the convolutional neural network model. Table 1 outlines the specific parameters of this model.

In Table 1, Conv2d represents standard convolution, bottleneck denotes the reverse residual structure, and avgpool signifies average pooled downsampling. Here, T is the expansion factor, C stands for the depth of the output characteristic matrix, n represents the frequency of the reverse residual structure’s usage in a given layer, and S indicates the step size of the first network layer in each stage. As evident from the table, the model heavily incorporates the reverse residual structure. The terminal convolution layer fulfills a similar function to a fully connected layer. The model’s final output characteristic matrix is measured. By applying the softmax classification layer to categorize the 1280-dimensional data, we can determine the recognition rate for seven distinct types of wheat grains.

The results of the MobileNet V2 network model recognition are presented in Figure 11. After five iterations on the training dataset, the model achieved a training accuracy exceeding 95% with rapid convergence. By the 50th iteration, the training accuracy remained above 99% while maintaining a loss under 0.1. When tested on the validation set, the model initially exhibited significant loss after 15 iterations but gradually stabilized after 35 iterations. Ultimately, after 50 iterations, the model achieved 97.5% accuracy with a loss hovering around 0.1.

Upon analyzing Figure 11, which compares the CNN and the MobileNet V2 network, several observations can be made. Within the training set, the CNN demonstrates relatively stable training accuracy and loss. The MobileNet V2 network exhibits a faster convergence speed, and after only two training sessions, the MobileNet V2 network achieves a recognition rate exceeding 90% in the training set.

In the test set, the CNN maintains a steady test loss of approximately 0.3. In contrast, the MobileNet V2 network experiences noticeable fluctuations in its test loss. Despite these variations, the MobileNet V2 network outperforms the CNN in terms of recognition rate. Specifically, compared to the CNN, the MobileNet V2 network boasts a 1.05% increase in accuracy within the training set and a 2.66% improvement in the verification set.

### 3.4. Subsection Comparison of the Three Modeling Results

According to the classification criteria, the CNN model and MobileNet V2 model were tested. In the test set, there were 50 grains of each of the 7 types of wheat, with a total of 350 grains. The correct number of grains identified by the three models for each of the seven types of wheat is shown in Table 2.

As evident from Table 2, the PSO-SVM network model demonstrates the lowest recognition accuracy among the seven wheat grain types, achieving a recognition rate of 93.17%. This is 1.43% lower than the CNN model’s performance. Notably, the recognition errors increased by 5, primarily due to an increase of 6 errors in the identification of insect-eaten grains. Conversely, the MobileNet V2 network model, built upon the foundation of the CNN, exhibits the highest recognition accuracy for all seven wheat grain types. It boasts a recognition rate of 97.71%, marking a 4% improvement over the PSO-SVM model and a 2.57% enhancement compared to the CNN model. The issue of higher recognition errors for insect-eaten grains has been addressed, and the recognition effect is the best among the three models.

The experimental results show that the MobileNet V2 model has the highest recognition rate, followed by the CNN model, and finally the PSO-SVM model. The image features learned by the MobileNet V2 Model Self-Learning can better represent the essential characteristics of wheat grains than the image features extracted manually.

## 4. Discussion

This study highlights the extensive potential of hyperspectral image technology in detecting and recognizing agricultural products. This technology offers notable benefits in identifying imperfect wheat grains by capturing the reflection or emission traits of objects across a continuous spectral range [24]. Furthermore, it enables non-contact and non-destructive testing, thus preventing secondary pollution or damage that may arise from conventional detection methods. This is crucial for maintaining the quality of agricultural products and ensuring food security.

In the study, a hyperspectral imaging system was constructed to capture visible near-infrared hyperspectral data for 2100 wheat grains, specifically targeting imperfect grains. The collected data underwent preprocessing with the Savitzky–Golay algorithm to eliminate noise and extract crucial spectral information, laying a strong foundation for further data analysis and model development. Additionally, the SPA was employed for hyperspectral data dimension reduction, which not only decreased computational demands but also preserved essential spectral characteristics, thereby enhancing the recognition model’s performance. This study conducted a comparative analysis of three distinct classification models (PSO-SVM, CNN, and MobileNet V2) in the context of recognizing imperfect grains of wheat. The findings reveal that MobileNet V2 exhibits the highest recognition efficiency, achieving a comprehensive recognition rate of 97.71%. This rate is significantly higher than those of the other two models. These results highlight the excellence of deep learning models, particularly lightweight networks like MobileNet V2, in handling intricate spectral data. Such networks offer not only a high recognition rate but also swift computational speed and reduced hardware demands, making them highly suitable for practical applications.

This technology demonstrates immense potential in areas such as agricultural product quality assessment, pest identification, and maturity evaluation [25]. Conventional agricultural product inspection techniques, including manual visual inspection [26] and chemical analysis [27], are not only time-consuming and labor-intensive but also relatively inefficient. When confronted with large-scale and high-frequency inspection demands, these traditional methods often struggle to cope. By integrating hyperspectral imaging technology with automated processing workflows and cutting-edge classification models [28,29], rapid scanning and data analysis of agricultural products become feasible, significantly enhancing inspection efficiency. For instance, this study used a hyperspectral imaging system to swiftly acquire extensive amounts of hyperspectral data on wheat grains. By utilizing an efficient classification model for real-time processing, it achieved the efficient identification of defective wheat grains. Moreover, this technology finds broad application in the quality inspection and classification of various agricultural products, enhancing the standardization and quality control in agricultural production and thereby promoting sustainable agricultural practices.

In summary, hyperspectral imaging technology offers distinct advantages in modern agriculture, providing crucial technical support for enhancing the efficiency of agricultural product quality monitoring, ensuring food safety, and promoting sustainable agricultural development.

## 5. Conclusions

(1) For the recognition of seven types of wheat grains, the PSO-SVM, CNN, and MobileNet V2 models achieved comprehensive recognition rates of 93.71%, 95.14%, and 97.71%, respectively, all surpassing 90%.

(2) In terms of recognition rates, the MobileNet V2 network outperforms the CNN. Specifically, MobileNet V2 boosts accuracy by 1.05% in the training set and by 2.66% in the verification set compared to the CNN.

(3) Generally, larger models with more parameters have the capability to capture more complex patterns in data, leading to better performance. However, smaller models can be more efficient in terms of computation and memory usage while still maintaining a certain level of performance. For instance, in this study, MobileNet V2 represents a lightweight model architecture with a relatively small size, yet it demonstrates the best performance in identifying imperfect wheat grains.

Thus, hyperspectral image technology combined with the classification model can be used to identify imperfect wheat grains effectively, which is a very effective method to improve the production of wheat seeds.

This study has made progress in identifying imperfect wheat grains using hyperspectral imaging technology, but there are still areas that are ripe for improvement: (1) Imperfect grains are not exclusive to wheat; they also occur in corn, soybeans, and other crops. Whether our hyperspectral imaging method for recognizing imperfect wheat grains can be applied to other grains remains to be seen, necessitating further investigation. (2) Due to time and resource constraints, this study did not establish a real-time detection system for imperfect wheat grains. We aim to develop such a system in the future. Additionally, given the high cost of hyperspectral cameras, we are exploring the use of a filter and CCD camera combination as a cost-effective alternative, capturing images specifically at the optimal spectral wavelength.

## Figures and Tables

**Table 1:** MobileNet V2 model parameters.

Input	Operator	t	c	n	s
1202 × a3	conv2d	-	32	1	2
602 × 32	bottleneck	1	16	1	1
602 × 16	bottleneck	6	24	2	2
302 × 24	bottleneck	6	32	3	2
152 × 32	bottleneck	6	64	4	2
82 × 64	bottleneck	6	96	3	1
82 × 96	bottleneck	6	460	3	2
42 × 160	bottleneck	6	320	1	1
42 × 320	conv2d1×1	-	1280	1	1
42 × 1280	avgpool7×7	-	-	1	-
1 × 1 × 1280	conv2d1×1	-	k	-	

**Table 2:** The number of correct identifications of three models.

Models	Broken	Germinated	Moldy	Insect-Eaten	Black Embryo	Gibberella	Perfect	Identification Rate
PSO-SVM	46	50	47	39	48	49	49	93.71%
CNN	48	49	48	45	48	47	48	95.14%
MobileNet V2	48	50	50	48	49	48	49	97.71%

## Data Availability

The data presented in this study are available upon request from the corresponding author. The data are not publicly available due to privacy concerns.
